# Genome Sequence of the Vancomycin-Producing *Amycolatopsis orientalis* subsp. *orientalis* Strain KCTC 9412^T^

**DOI:** 10.1128/genomeA.00408-13

**Published:** 2013-06-27

**Authors:** Haeyoung Jeong, Young Mi Sim, Hyun Ju Kim, Dong-Woo Lee, Si-Kyu Lim, Sang Jun Lee

**Affiliations:** Korean Bioinformation Center, Korea Research Institute of Bioscience and Biotechnology (KRIBB), Daejeon, Republic of Koreaa; Infection and Immunity Research Center, KRIBB, Daejeon, Republic of Koreab; School of Applied Biosciences, Kyungpook National University, Daegu, Republic of Koreac; GenoTech Corporation, Daejeon, Republic of Koread

## Abstract

*Amycolatopsis orientalis* is the producer of vancomycin, a glycopeptide antibiotic that is used for the treatment of serious infections with Gram-positive bacteria. Here we present the next-generation sequencing (NGS)-based 9.06-Mb draft genome sequence of the type strain *Amycolatopsis orientalis* subsp. *orientalis* KCTC 9412 (DSM 40040; ATCC 19795).

## GENOME ANNOUNCEMENT

*Amycolatopsis orientalis* is the producer of vancomycin (1), which is a glycopeptide antibiotic of last resort against methicillin-resistant *Staphylococcus aureus*, which causes serious clinical infections (2). For several decades, industrial strains have been developed through random mutagenesis or optimization of culture conditions (3) from *A. orientalis* subsp. *orientalis* KCTC 9412, the type strain of this species. However, genome sequence information for *A. orientalis* species is not yet publicly available. For the application of genome-scale metabolic engineering and synthetic biology to obtain higher yields of antibiotic production, we determined the genome sequence of the strain.

*A. orientalis* subsp. *orientalis* KCTC 9412^T^ was grown in tryptic soy broth containing wheat starch (0.1%) at 30°C. Cells were disrupted by lysozyme plus achromopeptidase (4), and chromosomal DNA was purified by a genomic DNA isolation kit (Promega). Genome sequencing was carried out using an Illumina HiSeq 2000 system. From the genomic library, 101-nucleotide paired-end reads (4.58 Gb, at 506× coverage) were produced. Pretreatment of reads and *de novo* assembly were done using the CLC Genomics Workbench ver. 6.0.1 (CLC bio). Quality-trimmed reads, consisting of 71.7% of initial bases, were assembled into 99 contigs (>200 bp, 69.0% G+C) using a word size of 64. Total contig length and *N*_50_ were 9,062,467 bp and 91,540 bp, respectively. The largest contig was 233,061 bp. We tested Velvet ver. 1.2.07 (5) and SOAPdenovo ver. 1.0.5 (http://soap.genomics.org.cn/) using a range of *k*-mers, but the CLC Genomics Workbench showed the best results in terms of *N*_50_.

The genome sequence was automatically annotated using the RAST server (6). It contains 8,249 protein-coding genes and 52 tRNA genes. Subsystem and clusters of orthologous groups (COG) coverages were 26.8% and 59.3%, respectively. Sequences related to the integrative plasmid pMEA100, which is known from *Amycolatopsis mediterranei* S699 (7), were not found. We identified a vancomycin biosynthetic gene cluster from the genome sequence, which was identical to the previously known gene cluster from *A. orientalis* ATCC 19795 (HE589771; 67,422 bp) at the nucleotide level, with an ~50-bp discrepancy in the intergenic region between *vpsA* and *vpsB*. We also sequenced the genomes of *A. orientalis* DSM 43388 and DSM 46075 and compared them with KCTC 9412^T^ sequences. Results obtained from average nucleotide identity (8) and ortholog clustering (9) suggested that the two DSM strains were very different from the type strain. In particular, among the eight nonribosomal peptide-synthetase (NRPS) gene clusters and two polyketide synthase gene clusters in KCTC 9412^T^, one NRPS gene homolog (>90% amino acid identity) was found in both DSM 43388 and DSM 46075. Vancomycin synthetic gene clusters were not found in the genomes of DSM 43388 and DSM 46075. However, we identified vancomycin resistance genes (10) *vanH, vanA*, and *vanX* in the genome of DSM 46075, which were also found in KCTC 9412^T^. Comparative genome analysis using all available *Amycolatopsis* species indicated that KCTC 9412^T^ is closest to *A. decaplanina* DSM 44594^T^.

### Nucleotide sequence accession numbers.

This Whole-Genome Shotgun project has been deposited at DDBJ/EMBL/GenBank under the accession number ASJB00000000. The version described in this paper is the first version, ASJB01000000.
